# Auditory scene analysis and sonified visual images. Does consonance negatively impact on object formation when using complex sonified stimuli?

**DOI:** 10.3389/fpsyg.2015.01522

**Published:** 2015-10-13

**Authors:** David J. Brown, Andrew J. R. Simpson, Michael J. Proulx

**Affiliations:** ^1^Crossmodal Cognition Lab, Department of Psychology, University of BathBath, UK; ^2^Biological and Experimental Psychology Group, School of Biological and Chemical Sciences, Queen Mary University of LondonLondon, UK; ^3^Centre for Vision, Speech and Signal Processing, University of SurreyGuildford, UK

**Keywords:** auditory scene analysis, consonance, signal complexity, blindness, cross-modal, sensory substitution

## Abstract

A critical task for the brain is the sensory representation and identification of perceptual objects in the world. When the visual sense is impaired, hearing and touch must take primary roles and in recent times compensatory techniques have been developed that employ the tactile or auditory system as a substitute for the visual system. Visual-to-auditory sonifications provide a complex, feature-based auditory representation that must be decoded and integrated into an object-based representation by the listener. However, we don’t yet know what role the auditory system plays in the object integration stage and whether the principles of auditory scene analysis apply. Here we used coarse sonified images in a two-tone discrimination task to test whether auditory feature-based representations of visual objects would be confounded when their features conflicted with the principles of auditory consonance. We found that listeners (*N* = 36) performed worse in an object recognition task when the auditory feature-based representation was harmonically consonant. We also found that this conflict was not negated with the provision of congruent audio–visual information. The findings suggest that early auditory processes of harmonic grouping dominate the object formation process and that the complexity of the signal, and additional sensory information have limited effect on this.

## Introduction

Our sensory systems provide a rich coherent representation of the world through the integration and discrimination of input from multiple sensory modalities (Spence, 2011). These low-level processes are modulated by high-order processing to selectively attend to task relevant stimuli. For example to attend to a speaker at a cocktail party we must select the low-level acoustic features that are relevant to the target, that is the person you are speaking with, from the environmental noise (Cherry, 1953). To accomplish this, feature-based sensory representations must be recombined into object-based representations in a rule based manner. In visual perception this is through scene analysis. Visual input is grouped into distinct objects based on Gestalt grouping rules such as feature proximity, similarity, continuity, closure, figure ground, and common fate (Driver and Baylis, 1989; Ben-Av et al., 1992). Similarly, there are rules that govern the arrangement of low-level stimuli into haptic and auditory objects. For the latter the process is called auditory scene analysis (ASA). Contrary to the spatial principles that guide visual categorization, grouping in ASA is at either a temporal or melodic level governed by proximity or similarity over time, pitch or loudness continuation, or at spectral levels including common fate, coherent changes in loudness, frequency, or harmony (Bregman, 1994).

While principles of ASA, such as frequency and harmony, may seem relatively unimportant to visual perception they hold relevance for rehabilitation techniques for the substitution of vision for the visually impaired (Proulx et al., 2008; Brown et al., 2011). Researchers have long strived to provide crucial visual information with compensatory techniques via alternate modalities such as touch – Braille, embossed maps, tactile sensory substitution – (Bach-y-Rita and Kercel, 2003; Rowell and Ungar, 2003; Jiménez et al., 2009) or more recently sound – auditory sensory substitution and auditory workspaces – (Frauenberger and Stockman, 2009; Abboud et al., 2014; MacDonald and Stockman, 2014). The conversion principles of sonification algorithms are not arbitrary but instead based on natural cross-modal correspondences and cross-modal plasticity (Frasnelli et al., 2011; Spence, 2011) which allow the coding of visual features (brightness, spatial location) into auditory ones (pitch, loudness, stereo pan). Sensory substitution devices go beyond simple feature detection, and are also effective in ‘visual’ tasks such as object recognition and localisation, and navigation (Auvray et al., 2007; Brown et al., 2011; Maidenbaum et al., 2013). Given that the substitution of vision by other sensory modalities can evoke activity in visual cortex (Renier et al., 2005; Amedi et al., 2007; Collignon et al., 2007), it is unclear whether the mechanisms of scene analysis are processed as visual objects or auditory objects. Is the grouping of feature-based sensory representations into auditory objects based on visual grouping principles or those of ASA?

It seems natural that if the signal is a sonification it would be processed as an auditory feature and therefore be subjected to grouping principles of ASA. However, with extensive research showing activation of ‘visual’ areas in response to ‘auditory’ stimulation (Amedi et al., 2007; Striem-Amit and Amedi, 2014) and visually impaired users defining information from sonifications as ‘visual’ (Ward and Meijer, 2010) it is important to ascertain whether or not the auditory characteristics are more salient to the final perception using sonifications rather than a straight extrapolation from the unimodal literature. There are certainly valid comparisons between the two modalities. For example, shape and contour are crucial for the organization and recognition of visual objects. In parallel the spectral and temporal contour of a sound, the envelope, is critical in recognizing and organizing auditory objects (Sharpee et al., 2011).

However, there are also critical differences. The output signal of the sonification algorithm is dependent of the visual properties of the stimulus and therefore can be a coarse representation relative to a controlled audio-only presentation. For example, the sonification of equal-width visual lines will have different frequency bandwidths dependent on the stimulus baseline on an exponential frequency scale – higher frequency baselines sonify to broader bandwidths, comprise of more sine waves, and are thus more complex than the sonification of an identical line lower down in the visual image. Thus, while the two pieces of visual information are perceived as having equivalent levels of complexity, there is variance between the complexities of the subsequent sonifications. Considering the purpose of sonifications is to convey visual information can we directly apply the principles of ASA, tested using auditory objects, to this?

If using the analog of two visual lines, equal in length (x-axis) but differing in elevation (y-axis), as two sonifications equal in duration (x-axis) but varying in baseline frequency (y-axis), we can apply ASA to make predictions on the mechanisms of feature segregation. Presented sequentially, with no requirement of identification (the two tones are separated in time), just noticeable differences (JND) in pitch should demonstrate low discrimination thresholds, typically between 1 and 190 Hz dependent on baseline frequency (Shower and Biddulph, 1931; Wever and Wedell, 1941). Presented concurrently, discrimination requires the identification of each tone based on the relative frequency components of each object. Considering this is one of the fundamental properties of the ear, the literature on this is scant. Thurlow and Bernstein (1957) reported two-tone discrimination at around 5% of the baseline frequency (at 4 kHz), while Plomp (1967), when assessing the ability to hear a harmonic in a harmonic complex, showed harmonic resolvability for five to seven lower harmonics. Plomp and Levelt (1965) evaluated explanations of consonance, that is the sensory experience of tonal fusion associated with isolated pairs of tones sharing simple frequency ratios, based on; frequency ratio, harmonic relationships, beats between harmonics, difference tones, and fusion. They concluded that the difference between consonant and dissonant intervals was related to the beats of adjacent partials, and that the transition range between these types of intervals were related to a critical bandwidth.

While this literature provides a solid grounding to predict results based on ASA it is important to note that in all these experiments the stimuli are generated as auditory objects, often with pure tones. This allows precision of the stimuli based on the exact auditory features you wish to test. For example, pure tones at specific frequencies can be used, or if testing the resolvability of harmonics complexes, tones with exact partials. Within the literature there appear to be no studies that contrast two-tone discrimination in which the precision of the stimuli is not controlled by auditory theory, as would be found when the signal is derived from visual features in a visual-to-auditory sonification. For example, with reference to the two line example above, would interval markers with varying complexity elicit similar results to what is found using controlled auditory stimuli? With this is mind we evaluated the segregation of two ‘auditory’ signals sonified from two equal length parallel lines at varying intervals. In a simple 2AFC paradigm the listener was required to indicate their perception of ‘one-ness’ or ‘two-ness’ in presented tonal complexes(Thurlow and Bernstein, 1957; Kleczkowski and Pluta, 2012). Based on the auditory literature we hypothesized that segregation of the two lines into separate objects would be problematic when the sonifications had consonant harmonic relations.

In a second part of the experiment we used a multisensory paradigm to evaluate whether any influence in discrimination, due to ASA rules, could be negated by the provision of additional information in another modality. Our rationale and methodology were simple. Extensive research has demonstrated the efficacy of using multisensory, rather than uni-modal stimuli, with audio–visual information shown to enhance visual perception (Frassinetti et al., 2002) visual search (Iordanescu et al., 2008) and increase performance in spatial and temporal tasks. In speeded classification (SC) paradigms (Evans and Treisman, 2010) in which participants have to rapidly discriminate visual targets while presented with task irrelevant auditory stimuli, response times increase and accuracy decreases if the auditory stimulus is incongruous, i.e., high visual elevation paired with low pitch tone (Bernstein and Edelstein, 1971; Marks, 1974; Ben-Artzi and Marks, 1995).

Crucial in multisensory integration is the binding of the unimodal stimuli into one perceived event based on: low-level spatial and temporal synchrony (Spence, 2011), temporal correlation (Radeau and Bertelson, 1987; Recanzone, 2003), or top down cognitive factors such as semantic congruency (Laurienti et al., 2004). For example, incongruent audio–visual spatial information shows a localisation bias toward visual information, in the ventriloquist effect, even when cued to the auditory stimulus (Bermant and Welch, 1976; Bertelson and Radeau, 1981) while separation of asynchronous audio–visual stimuli was perceived as shorter if presented in congruent rather than incongruent spatial locations (Soto-Faraco et al., 2002; Vroomen and de Gelder, 2003) with the auditory information appearing to dominate (Fendrich and Corballis, 2001; Soto-Faraco et al., 2004).

Considering this we manipulated the first task by providing either congruent multisensory stimuli, in which the sonification and visual presentation were associated (e.g., two-tone sonification and two visual lines) or incongruent (e.g., two-tone sonification and one visual line) to the listener. The task requirements were as before with the listener instructed to indicate how many visual lines had been sonified to create the stimulus. Based on the multisensory literature, we hypothesized that congruent audio–visual stimuli would facilitate superior performance in contrast to performance with both incongruent audio–visual and audio only stimuli.

## Materials and Methods

### Participants

We recruited 36 participants (28 female) via an Undergraduate Research Assistant module. Participant age ranged from 18 to 25 years old (*M* = 20.17, *SD* = 1.30). All participants provided informed written consent, and had normal or corrected eyesight, normal hearing and educated to undergraduate level. Four participants self-reported as left handed and all were naïve to the principles of sonification. 12 participants didn’t return for the second part of the study and this is reflected in the analysis. The study was approved by the University of Bath Psychology Ethics Committee (#13-204).

### Materials and Stimulus Design

Visual stimuli were created in Adobe Photoshop 3.0 with the sonifications using the principles of The vOICe (Meijer, 1992) algorithm. Frequency analysis of the sonifications was conducted in Cool Edit Pro 2.0 with all visual stimuli and sonifications presented in E-Prime 2.0 running on a Windows 7 PC. Sonifications were transmitted to the listener via Sennheiser HD 585 headphones. All statistical analysis was conducted using SPSS version 21.0.

### Stimulus Design

**FIGURE 1:**
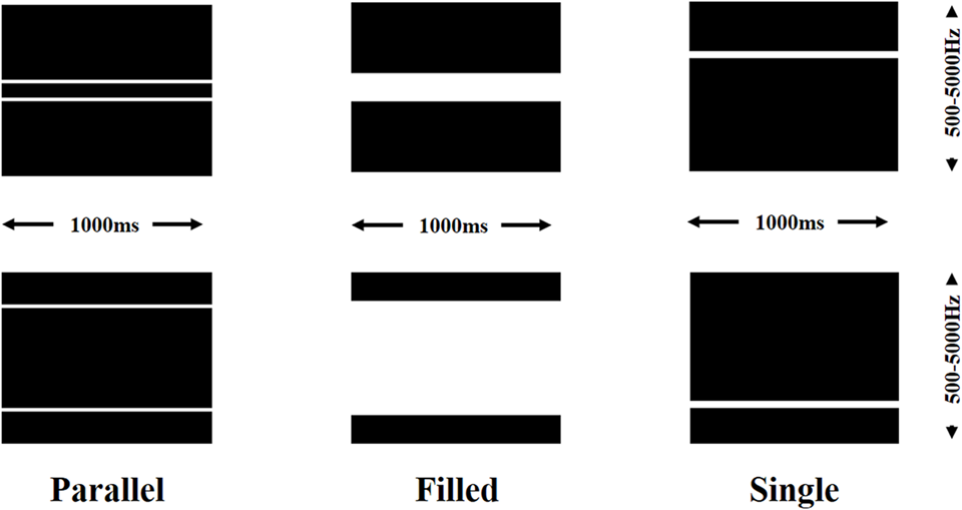
**Illustration of the types of visual stimuli used for sonification.** Two examples shown of parallel lines with different intervals, filled lines with different bandwidths, and single lines at different frequencies. Duration and frequency range of the sonifications also shown.

In Photoshop a grid of 48 pixel × 1.5 pixel rows was overlaid on a black background. Solid white lines were drawn over the full x-axis of the background with width and interval dependent on the stimulus type. Example of each type of line can be seen in **Figure 1**. For the parallel line stimuli two one-row lines, separated by the designated interval were created. The interval was varied from a two-row interval to a 42 row interval, with each interval gap increasing by two rows. The initial starting point was the center of the y-axis with each interval involving moving the top line up one row and the bottom line down 1 row from baseline or the previous stimulus. There were two types of single line stimuli. Filled stimuli took the parallel line stimuli and filled the gap between the two lines with white pixels. Thus the top and bottom lines were the same as the parallel line counterparts but with no interval between. The single line stimuli consisted of a line 2 rows thick (giving the same amount of white pixels as the parallel line). In total there were 23 parallel line, 24 single, and 24 filled stimulus images (two lines together at the central point of the y-axis was classified as a single line).

The lines were sonified using the following principles: the duration of each sonification, represented on the x-axis, was consistent for all stimuli (1000 ms), pitch was mapped to the y-axis with a range of 500 Hz (bottom) to 5000 Hz (top).White pixels were sonified at maximum volume (-65 dB) with black pixels silent. Each sonification therefore comprised of two complex tones at varying frequencies playing concurrently for 1000 ms (parallel lines), or one complex tone with the same top and bottom frequencies as the parallel line counterpart playing for 1000 ms (filled lines), or one complex tone at a consistent ‘visual’ width playing for 1000 ms (single line). Parallel line sonifications were categorized as consonant or dissonant based on the frequency range of the interval between the two lines.

### Procedure

Participants watched a PowerPoint presentation with audio–visual examples of the sonification process with a brief introduction to its applications. Example parallel lines, plus the two types of single lines with their sonifications were included as well as an example of the task procedure. For each trial of the main task the listener was presented with a soundscape which had been sonified from either 1 or 2 visual lines. Their task was to indicate on the PC keyboard whether the sonification was of 1 or 2 lines. Participants were explicitly told in both the instructions and PowerPoint that a filled line was classed as a single line. There was no visual information or post-trial feedback given. Each experimental block consisted of 96 trials (48 (2 × 24) × parallel, 24 × filled, 24 × single) with trial order fully randomized within block and no repeated trials. There were four blocks in total, randomized across participants, to give 386 trials in total.

**FIGURE 2:**
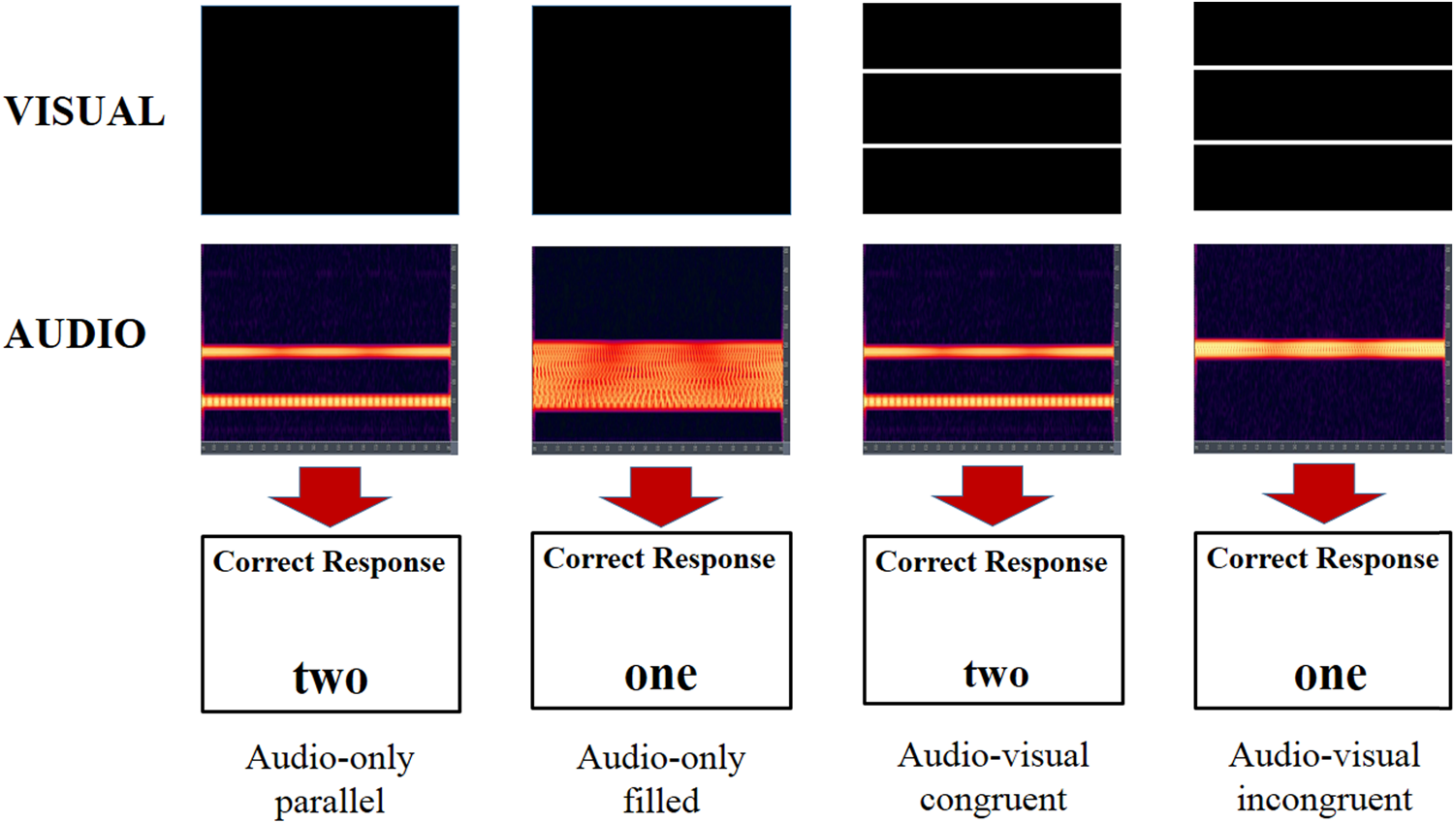
**Illustration of four different trial types.** What the participant sees on screen is shown in the top row. Spectrographs of the audio signal the participant hears is shown in the second row with the correct response in the third row. The two trials on the left are audio-only trials with the two on the right congruent and incongruent audio–visual trials.

The audio–visual task had the same listener requirements as the audio-only task, that is, to indicate how many lines were used to create the sonification. For each trial the listener heard a soundscape sonified from one or two lines. At the same time an image of one or two white lines appeared on the PC monitor. The audio–visual presentation could either be congruent, where the number of lines matched over both modalities, or incongruent where there was a mismatch. The participants were informed that while it was a requisite to look at the screen for timing purposes they were not required to indicate how many visual lines they perceived, just the number of ‘lines’ in the soundscape. As with the audio-only task there was no feedback. Again there were 4 blocks of 96 randomized trials. Examples of the example trials in both conditions are shown in **Figure 2**.

## Results

**FIGURE 3:**
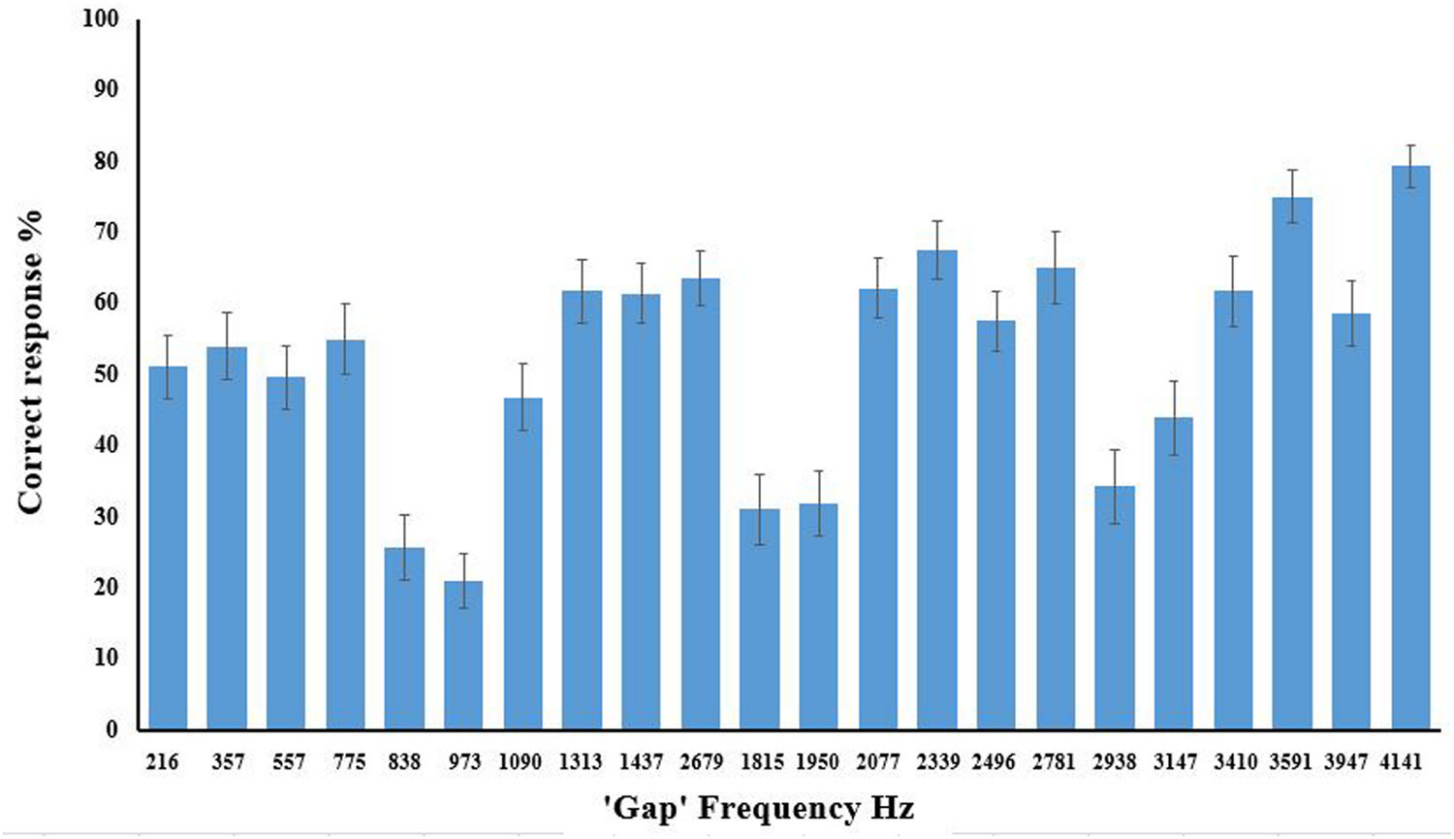
**Correct response (%) for parallel line stimuli for each frequency gap prior to categorization into consonant and dissonant groups.** Error bars show ±1 SEM.

**FIGURE 4:**
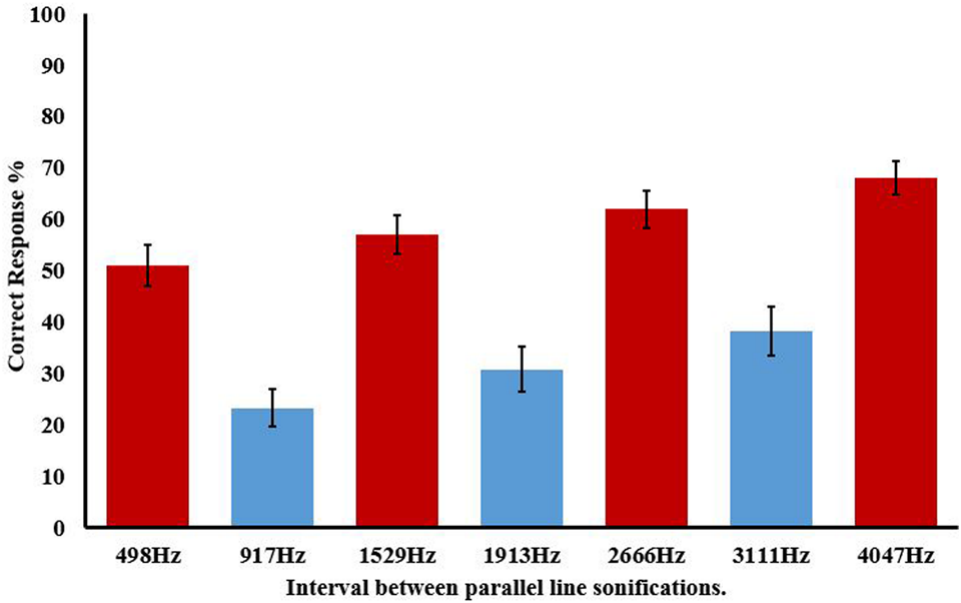
**Correct response (%) for parallel line discrimination with after categorization into consonant (blue) and dissonant (red) groups.** Frequency ranges for each interval are shown on the x-axis. Error bars show ±1 SEM.

Consider accuracy for the parallel line condition first. **Figure 3** displays accuracy for individual parallel line frequencies, and clearly illustrates that the size of the interval between lines affects accurate recognition [*F*(8.52,298.04) = 21.937, *p* < 0.0005, $\eta_{p}^{2}$ = 0.385]. It is also clear that this cannot be solely due to proximity as some proximal lines (e.g., 498 Hz) are discriminated better than more distal lines (e.g., 3111 Hz), indicating that the predicted harmonic grouping is the relevant factor. **Figure 3** also displays the pattern for consonant (<50%) and dissonant (>50%) stimuli which matches the predictions from the categorization based on consonance and dissonance. Analysis of variance on these seven groups, as shown in **Figure 4**, again showed a main omnibus effect [*F*(3.19,111.52) = 42.182, *p* < 0.0001, $\eta_{p}^{2}$ = 0.547].

**FIGURE 5:**
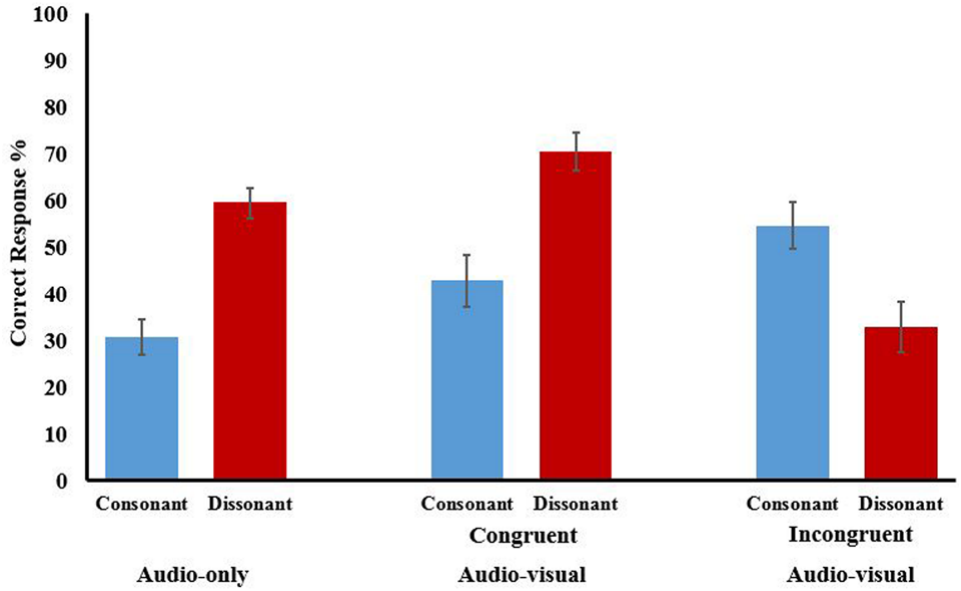
**Correct response (%) for parallel line discrimination when trials used congruent and incongruent audio–visual stimuli.** Means are shown for consonant (blue) and dissonant (red) intervals for audio-only and audio–visual conditions. Error bars show ±1 SEM.

With harmonicity appearing the main factor in parallel line discrimination all relevant conditions were analyzed together: audio-only consonant, audio-only dissonant, audio–visual consonant congruent, audio–visual consonant incongruent, audio–visual dissonant congruent, and audio–visual dissonant incongruent. Results are shown in **Figure 5** and **Table 1**. With accuracy as the D.V., an ANOVA, Greenhouse-Geisser corrected for violation of sphericity (𝜀 = 0.588), showed an omnibus main effect [*F*(2.94,64.69) = 19.162, *p* < 0.000, $\eta_{p}^{2}$ = 0.466] again displaying that, when factoring in audio–visual conditions, the size of the interval between parallel lines is influential in line discrimination. To assess where these differences lay planned contrasts, Bonferroni corrected for multiple comparisons, were conducted.

**Table 1:** Correct response (%), for parallel line discrimination for consonant and dissonant stimuli in; audio-only, congruent audio–visual, and incongruent audio–visual conditions.

	Consonant		Dissonant	
	Mean	*SD*	Mean	*SD*
Audio-only	30.73	22.86	59.48	19.54
**Audio–visual**				
Congruent	42.75	26.33	70.58	19.33
Incongruent	32.79	25.85	54.55	24.16


For trials where the stimuli were audio-only harmonicity had a large impact. Dissonant stimuli (*M* = 59.48), where the interval should not elicit any tonal confusion, were discriminated more successfully than consonant stimuli (*M* = 30.73) where harmonic relations should impact on performance [*MD* = 27.525, 95% CI(15.84,39.21), *p* < 0.0005]. The latter were also significantly below what would be expected by chance [*t*(35) = -5.058, *p* < 0.0005, *d* = 1.34] illustrating the magnitude of the ‘confusion’ caused by these harmonic relations.

Could this effect be anyway negated by using multisensory stimuli providing additional visual information? With the literature implying that multisensory binding requires some form of synchronicity we would only expect improved performance for audio–visual trials that were congruent, that is, provide the same line information via different modalities. The contrasts for the consonant stimuli showed no evidence of increased performance due to either congruent (*M* = 42.75) or incongruent (*M* = 32.79) audio–visual stimuli with significance levels of *p* = 0.797 and *p* = 0.984, respectively.

For dissonant stimuli, where performance in the audio-only condition was already significantly above chance [*t*(35) = 2.912, *p* = 0.006, *d* = 3.04] with no issues of harmonic relations we would expect an improvement in performance congruent trials in the audio–visual conditions. While the contrasts showed higher mean accuracy for the congruent condition (*M* = 70.58) and a lower one for the incongruent (*M* = 54.55), compared to the audio-only (*M* = 59.95) neither differences were significant with *p*-values of 0.445 and 0.984, respectively.

Secondly we considered whether proximity was an influence on discrimination of parallel lines, that is, would sonified lines closer together be less likely to be segregated into separate objects? Looking at the seven groups categorized by the frequency ranges shown in **Figure 4**, we only contrasted within groups, that is, consonant versus consonant and dissonant versus dissonant. With the harmonicity effect having such a profound effect on performance comparisons between consonant and dissonant groups would naturally show a significant effect with the variance explained by these harmonic relations.

With accuracy as the dependent variable an ANOVA factoring in all consonant groups (audio-only, audio–visual congruent and audio–visual incongruent) showed an omnibus main effect for proximity [*F*(8,176) = 3.528, *p* = 0.001, $\eta_{p}^{2}$ = 0.138] with a separate ANOVA for dissonant groups showing similar [*F*(11,242) = 5.335, *p* = 0.001, $\eta_{p}^{2}$ = 0.195]. The Bonferroni corrected planned contrasts for both analyses tell a similar story. The only significant planned contrasts were between the congruent and incongruent audio–visual categories. For example, for consonant trials disregarding harmonicity, discrimination in the largest congruent category was better than for the two smallest incongruent categories (*p* = 0.008) and (*p* = 0.013), respectively. Dissonant trials in the smallest congruent group were better than for the smallest (*p* = 0.002) and second smallest (*p* = 0.018) incongruent groups. The second largest congruent elicited better scores than all four incongruent groups (smallest-to-largest, *p* = 0.026, *p* = 0.001, *p* = 0.008, *p* = 0.001), with the largest congruent group better than the smallest (*p* = 0.009) and largest (*p* = 0.009) incongruent. There were no significant contrasts within groups or involving the audio-only trials.

Analysis of the filled line data corroborates the lack of any effect of proximity. These lines retained the same top and bottom frequencies as the parallel lines but with the interval filled with white pixels/sonified noise. Without the intervals there can be no effect of harmonicity and therefore any differences are due to proximity or signal bandwidth. With all groups (7 × audio-only, 7 × audio–visual congruent, 7 × audio–visual incongruent) entered into an ANOVA there was a significant omnibus main effect [*F*(20,360) = 3.401, *p* < 0.0005, $\eta_{p}^{2}$ = 0.159]. However, while there were 17 significant contrasts at an alpha of <0.05 these were all between audio–visual congruent (good) and incongruent groups (poor) with no differences within groups or involving the audio-only condition.

In summary. When presented with audio-only stimuli where the interval had no harmonic relations the task was relatively easy with participants scoring above chance. However, when the interval does have harmonic relations, signified by tonal-fusion, the negative impact of this makes the task difficult with participants below chance levels. The use of audio–visual stimuli has little impact on lessening the effect of harmonicity and even when this effect is discounted, i.e., dissonant stimuli only, the congruent trials show a trend of better discrimination, but not reaching significance. Secondly, there is little evidence that proximity influences the discrimination of the sonifications with the only effects in this analysis being down to the use of congruent and incongruent audio–visual stimuli.

## Discussion

In this study we evaluated whether feature segregation of sonified horizontal lines would be influenced by rules of ASA. Unlike simple stimuli used in auditory research, the sonifications here were complex, with wider interval marker bandwidths dictated by the visual features of the stimulus interacting with the principles of the sonification algorithm. However, even with this coarse representation, sonifications with consonant intervals demonstrated poor segregation as predicted by ASA. Secondly we assessed whether the provision of additional multisensory information would negate the effects of harmonicity. While congruent audio–visual information displayed a trend for superior feature segregation, relative to incongruent audio–visual and audio-only, this only reached significance for the former contrast.

The results fall broadly in line with what is predicted in the auditory literature (Plomp and Levelt, 1965; Bregman et al., 1990; Bregman, 1994) demonstrating the negative impact of consonance on feature segregation. Even when visual lines were almost the full height (y-axis) of the workspace apart, with associated sonifications separated by >3100 hz, harmonic relations elicited the perception of one object. While these findings are not too surprising they do emphasize the robustness of the effect to interval markers of varying complexity. The logarithmic frequency conversion of the algorithm renders visual lines of equal width as sonifications whose bandwidths are dependent on their elevation in the visual field. For example, in our study the frequency bandwidth of a two-pixel wide line at the top of the screen was over 800 hz greater than the equivalent line at the bottom of the screen. Within the somewhat sparse simultaneous two-tone discrimination literature in the auditory domain, in which visual factors are not applicable, this interval marker bandwidth variability is not assessed as stimuli parameters can be more controlled. Of course it would be interesting to evaluate how much variance between the two markers, in bandwidth and other features, would be required to reduce the consonance effect. There is certainly evidence that two-tone complexes are more easily resolved if the amplitude of one of the tones is more intense (Arehart and Rosengard, 1999) and this could have been evaluated in the present experiment by manipulating the shading of one of the visual lines.

Using The vOICe algorithm for the visual-to-auditory conversion necessitates a signal that is not static in the stereo field over time, that is, the signal initiates in the left headphone and pans across the stereo field to the right headphone over the duration of the scan. In a simultaneous two-tone pitch discrimination task Thurlow and Bernstein (1957) compared conditions where either the two tones were presented to the same ear (analogous to the present study), or presented to separate ears. Results showed little difference in discrimination for the five tested frequency levels when led to separate ears, however, when led to the same ear equivalent performance was only for stimuli where masking effects were minimized. If The vOICe signal was led to separate ears with the low frequency line scanning right-to-left and the high frequency line left-to-right, would this negate the masking effects demonstrated in the study? It is certainly a consideration for future research.

Simultaneous two-tone discrimination has been evaluated in different users to assess individual and group differences. An obvious group to test is trained musicians as successful pitch discrimination is an essential tool in their skillset. Kleczkowski and Pluta (2012) demonstrated that trained musicians were able to discriminate pitches at narrower levels than non-musicians, with similar results for musicians resolving harmonics in inharmonic complexes (Plomp, 1976). Musicians have also shown higher levels of performance using sensory substitution devices with Haigh et al. (2013) reporting musical ability correlating with higher acuity in a task using the vOICe and the Snellen Tumbling ‘E’. All participants in the study were sighted and naïve to sensory substitution and yet demonstrated acuity approaching the legal blindness definition of 20/200. In a similar acuity test with blind participants trained to use the device even lower acuity was reported (Striem-Amit et al., 2012) illustrating not only the effect of training but also potentialities due to superior auditory abilities, such as frequency discrimination (Roder et al., 1999; Wan et al., 2010), posited to be found in these populations. It would therefore be of great interest to test whether highly trained blind users of The vOICe could overcome the effect of consonance found in the present study. If so, this psychophysical test will provide solid evidence whether, through perceptual learning, the user is truly ‘seeing’ the sound or just hearing it. Considering the strength of consonance reported, it is highly doubtful that the effect would be negated in auditory domain and thus any difference in performance in these populations would imply a percept beyond audition.

The strength of the consonance effect is further exemplified by the limited influence of congruent and incongruent visual information. In speeded classification tasks evaluating cross-modal congruency, classification of visual stimuli as ‘high’ or ‘low’ has been shown to be more rapid if accompanied by tones that were congruent rather than incongruent (Bernstein and Edelstein, 1971; Ben-Artzi and Marks, 1995) with Evans and Treisman (2010) showing that cross-modal mappings between audio and visual stimuli are automatic and affect performance even when irrelevant to the task. This integration of temporally synchronous multisensory information is weighted to specific modalities as a function of the task (Spence, 2011), drawing support from a metamodal theory of the brain organization (Pascual-Leone and Hamilton, 2001). Here the brain is viewed as a task based machine with brain areas that are functionally optimal for particular computations; auditory areas for temporal tasks and visual for spatial (Proulx et al., 2014). In the present study the discrimination task can be considered spatial as the temporal features of the stimuli were identical. True to the metamodal theory, this adds weight to the visual information. If the audio–visual stimuli were congruent this should elicit better performance, and while the data showed a trend for this, it was not strong enough to bring discrimination of consonant stimuli above chance levels. Conversely, the incongruent visual information should reduce performance as there is extra weight attributed to the irrelevant distractor but again this trend was non-significant. Naturally with no access to visual information the blind users would not experience this audio–visual congruence, however, this could be tested using congruent and incongruent tactile stimuli. Simple methods such as embossed print outs of the visual workspace, or more technological based techniques involving haptic displays could be utilized to give multisensory information.

The results of our experiment show that the influence of consonance on object segregation is applicable to the sonification of coarse visual objects, but how can this information be suitably utilized? One approach to sonify a visual computer workspace is to evaluate the original visual stimulus and a spectrograph of it. Comparing these to the auditory representation would allow an evaluation of any potential auditory masking that might arise. This could include the direct mapping of spectrographs over the visual workspace in the development stage. Secondly, it would be interesting to evaluate how much consonance impacts on the use of sensory substitution devices when used in real-time. In such scenarios the sonified visual field updates at the device scan rate (1000 ms at default) to provide a continuous stream of ‘static’ frames. Thus, two parallel line sonifications masked in the first frame would only remain masked in the following frame if the device sensor, and background, remained static. For example, if the sensor was closer to the object in the second frame the parallel lines would be more disparate on the y-axis, the auditory interval increased, and the consonance negated.

A second consideration is variability and density of information provided in real-time device use. The present study utilized relatively simple stimuli, equal in all properties aside from auditory frequency, on a silent background. Objects encountered in everyday use are likely to be considerably more complex and therefore, even with masking, there should be sufficient unmasked signal to facilitate recognition. Indeed in a simple object recognition task using The vOICe, Brown et al. (2014) demonstrated equitable performance for degraded signals with limited information in contrast to more detailed stimuli.

Considering the above it seems unlikely that the negative effects of consonance would impact on real-time use of sensory substitution devices, although it should be considered if using static objects in early training paradigms. Interestingly, however, reducing dissonance has already been applied to visual-to-auditory sensory substitution. The EyeMusic uses similar conversion principles to the vOICe as well as coding basic colors to musical instruments (Abboud et al., 2014). In an attempt to make device use less uncomfortable, a pentatonic scale, alongside a reduced frequency range, is used to reduce dissonance. This is logical considering dissonance in audition is associated with a harsh perceptual experience. However, as we have demonstrated in our simple object discrimination task, dissonance appears important in feature segregation and it may be worth evaluating if there would be a comfort-function trade off in such tasks using EyeMusic.

## Conflict of Interest Statement

The authors declare that the research was conducted in the absence of any commercial or financial relationships that could be construed as a potential conflict of interest.
